# Crystal structure of a covalently linked Aurora-A–MYCN complex

**DOI:** 10.1107/S2059798322011433

**Published:** 2023-01-01

**Authors:** Mathias Diebold, Lars Schönemann, Martin Eilers, Christoph Sotriffer, Hermann Schindelin

**Affiliations:** a Institute of Pharmacy and Food Chemistry, University of Würzburg, Am Hubland, 97074 Würzburg, Germany; b Institute of Structural Biology, Rudolf Virchow Center for Integrative and Translational Bioimaging, University of Würzburg, Haus D15, Josef-Schneider-Strasse 2, 97080 Würzburg, Germany; c Theodor-Boveri-Institute, Biocenter, University of Würzburg, Am Hubland, 97074 Würzburg, Germany; F. Hoffmann-La Roche Ltd, Switzerland

**Keywords:** Aurora-A, MYCN, cross-linking, neuroblastoma cells, oncogenic transcription factors, cancer targets

## Abstract

Formation of the Aurora-A–MYCN complex increases levels of the oncogenic transcription factor MYCN in neuroblastoma cells by abrogating its degradation through the ubiquitin proteasome system. While some small-molecule inhibitors of Aurora-A were shown to destabilize MYCN, clinical trials have not been satisfactory to date. MYCN itself is considered to be ‘undruggable’ due to its large intrinsically disordered regions. Targeting the Aurora-A–MYCN complex rather than Aurora-A or MYCN alone will open new possibilities for drug development and screening campaigns. To overcome the challenges that a ternary system composed of Aurora-A, MYCN and a small molecule entails, a covalently cross-linked construct of the Aurora-A–MYCN complex was designed, expressed and characterized, thus enabling screening and design campaigns to identify selective binders.

## Abstract 2

The crystal structure of a covalently linked Aurora-A–MYCN complex is reported, enabling drug design and screening studies.

## 1. Introduction

Aurora-A is a serine/threonine kinase that belongs to the subfamily of Aurora kinases, which consists of Aurora-A, Aurora-B and Aurora-C. The three kinases are closely related; in their catalytic domains, Aurora-B and Aurora-C share 71% identity with Aurora-A. All three kinases play important but different roles during mitosis, which is presumably due to the fact that their N-terminal domains differ both in length and in sequence (Carmena & Earnshaw, 2003 ▸; Katayama *et al.*, 2003 ▸). Aurora-A is a viable cancer target, and its overexpression has been described to be a negative prognostic factor in neuroblastoma patients (Shang *et al.*, 2009 ▸). The protein–protein complex formed between the kinase Aurora-A and the oncogenic transcription factor MYCN antagonizes proteasomal degradation of MYCN in neuroblastoma cells (Otto *et al.*, 2009 ▸). Formation of this complex is confined to the S phase of the cell cycle (Büchel *et al.*, 2017 ▸), while, for example, the native activator TPX2 binds to Aurora-A during the G2/M phase (Hsu *et al.*, 2017 ▸).

The proto-oncogenic transcription factor MYCN, together with MYCL and MYC, is part of the MYC family of proteins, which interact with virtually all active promoters to regulate gene expression and globally maintain genome stability (Baluapuri *et al.*, 2020 ▸). The degradation of MYC proteins through the ubiquitin proteasome system is regulated by phosphorylation, which induces polyubiquitylation through the E3 ligase FBW7 (Adhikary & Eilers, 2005 ▸). Regulation of the levels of MYC family members is an ongoing area of research, including the recent discovery that MYC is regulated through two degrons at Thr58 and Thr244. Together, they recruit an FBW7 dimer followed by assembly of the SCF E3 ubiquitin ligase complex, resulting in polyubiquitylation (Welcker *et al.*, 2022 ▸). MYCN, however, lacks the second degron and relies on the degron at Thr58 for degradation by FBW7. Complex formation between Aurora-A and MYCN involving a site in MYCN in close proximity to the Thr58 degron abrogates FBW7 binding to the transcription factor and hence stabilizes MYCN levels.

Some inhibitors of Aurora-A can prevent Aurora-A from stabilizing MYCN through allosteric effects, thereby destabilizing the Aurora-A–MYCN interaction (Brockmann *et al.*, 2013 ▸; Gustafson *et al.*, 2014 ▸). However, clinical trials with one of these compounds, alisertib, targeting childhood neuroblastoma, did not yield satisfactory results (Mossé *et al.*, 2019 ▸). MYCN itself is considered to be ‘undruggable’ by small molecules, mostly because of its large stretches of intrinsically disordered residues (Wolpaw *et al.*, 2021 ▸). Therefore, targeting the complex formed by Aurora-A and MYCN directly could present a valuable strategy to modulate MYCN levels in cancer cells. Since drug-design efforts rely on the experimental validation of compounds developed *in silico* and the derived structure–activity relationships of possible candidate molecules, we designed, expressed and characterized a covalently linked complex between Aurora-A and MYCN. By engineering a stable binary protein–protein complex, future biophysical experiments will not need to account for the formation of the ternary protein–protein–small molecule complex, but can instead focus on the interactions between the candidate small molecules and the protein–protein complex.

## 2. Materials and methods

### 2.1. Production and purification of the Aurora-A–MYCN cross-linked complex (ANX)

The sequence of truncated human Aurora-A encompassing residues 122–403 with three mutations, C290A, K339C and C393A, was cloned into an NcoI/NotI-opened pETM11 vector. The protein was expressed with an N-terminal His_6_-TEV tag in *Escherichia coli* BL21(DE3) pLysS cells (Novagen). The cells were grown at 37°C to an OD_600_ of 0.6 and protein expression was induced with 0.5 m*M* isopropyl β-d-1-thio­galactopyranoside, followed by incubation for 16 h at 18°C. The cells were harvested by centrifugation at 6000*g* for 20 min.

For purification, the cell pellets were resuspended in lysis buffer [200 m*M* NaCl, 50 m*M* HEPES pH 7.5, 5 m*M* MgCl_2_, 10% glycerol, 20 m*M* imidazole, 0.02% NP-40, complete EDTA-free protease-inhibitor cocktail (Roche); ∼10 ml lysis buffer per gram of cell pellet] and lysed by sonication on ice with five 1 min pulses at maximal amplitude on a LABSONIC P sonifier (B. Braun Biotech International). The lysate was cleared by centrifugation (30 min at 30 000*g* and 4°C) before being loaded onto a 5 ml HisTrap HP column (GE) for IMAC purification. The column was washed with 20 column volumes of lysis buffer and Aurora-A was eluted with a linear imidazole gradient (200 m*M* NaCl, 50 m*M* HEPES pH 7.5, 5 m*M* MgCl_2_, 10% glycerol, 20–500 m*M* imidazole) over 20 column volumes. Aurora-A-containing fractions were pooled and concentrated approximately twofold (Amicon Ultra-15, molecular-weight cutoff 10 kDa) before further purification by size-exclusion chromatography (SEC) on a HiLoad Superdex 200 26/600 pg column in 200 m*M* NaCl, 20 m*M* HEPES pH 7.5, 5 m*M* MgCl_2_, 10% glycerol, 1 m*M* tris(2-carboxyethyl)phosphine (TCEP). Fractions containing Aurora-A were pooled, resulting in a protein concentration of 0.78 mg ml^−1^ as determined by the absorption at 280 nm using a calculated molar extinction coefficient of 41 040 *M* ^−1^ cm^−1^. The purified protein was shock-frozen in liquid nitrogen and stored at −80°C.

Prior to cross-linking, Aurora-A was diluted to 20 µ*M* with cross-linking buffer (200 m*M* NaCl, 100 m*M* K_2_HPO_4_ pH 7.0, 10% glycerol, 1 m*M* EDTA) and incubated with 5 m*M* TCEP (from a 100 m*M* stock in 1 *M* Tris–HCl pH 8.0) at room temperature for 30 min. The reduced protein was washed three times by twofold concentration (Amicon Ultra-15, molecular-weight cutoff 10 kDa) and subsequent replenishment with cross-linking buffer to the original volume.

The peptide corresponding to residues 28–89 of MYCN, in which Asn85 was replaced with a lysine containing a maleimidopropionic acid linker on its amino group (Lys-malpropyl), was custom-synthesized by Peptide Synthetics (Peptide Protein Research, UK). The resulting material was dissolved in pure DMSO to a concentration of 10 mg ml^−1^ as determined by the absorption at 280 nm using a calculated molar extinction coefficient of 19 630 *M* ^−1^ cm^−1^.

For cross-linking, reduced and buffer-exchanged Aurora-A was mixed with a twofold molar excess of the MYCN peptide and incubated for 4 h at room temperature. The cross-linking reaction was stopped by the addition of 10 m*M* DTT and incubation for an additional 15 min at room temperature.

**Figure 1:**
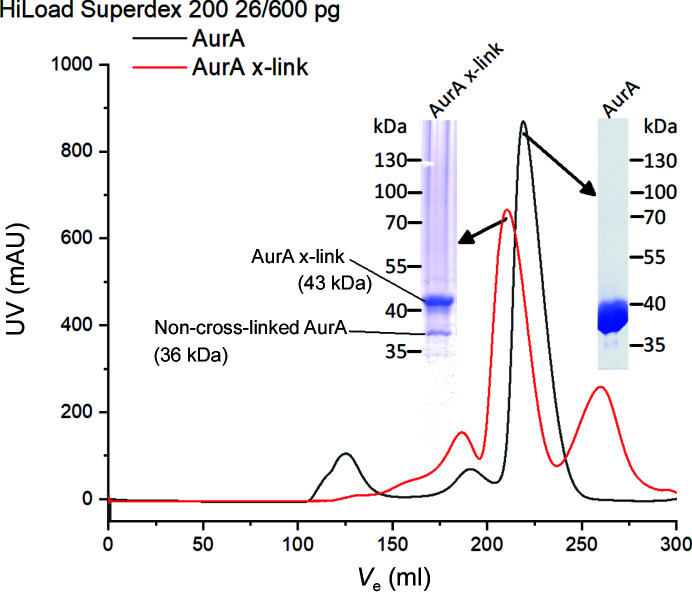
Verification of the Aurora-A cross-link by SEC and SDS–PAGE analysis. Chromatograms of Aurora-A alone (AurA; black) and Aurora-A after cross-linking (AurA x-link; red) are shown. The respective peak fraction was analyzed by SDS–PAGE with Coomassie Brilliant Blue staining.

The cross-linked reaction product was concentrated about twofold and purified by SEC (HiLoad Superdex 75 16/600 pg) in 200 m*M* NaCl, 100 m*M* K_2_HPO_4_ pH 7.0, 10% glycerol, 5 m*M* MgCl_2_. Fractions were pooled and concentrated to 0.56 mg ml^−1^ (13 µ*M*; Fig. 1 ▸). For crystallization, the cross-linked construct was rebuffered into Bicine buffer (100 m*M* Bicine pH 9, 250 m*M* NaCl, 5 m*M* MgCl_2_, 10% glycerol) and concentrated to 3.44 mg ml^−1^. Macromolecule-production information is summarized in Table 1 ▸.

### 2.2. Crystallization

Several crystallization screens were performed using the Bicine-buffered ANX construct both in the presence and absence of ADP. For screens containing ADP, 100 µl ANX was mixed with 5 µl of a 500 m*M* ADP solution and incubated at room temperature for 1 h. Crystallization screens were pipetted using three-drop Intelli-Plates (Art Robbins Instruments) with 50 µl reservoir solution and drops consisting of 200 nl reservoir solution mixed with 200 nl protein solution. In the absence of ADP, the screens employed were Optimix4PEG (Fluidigm), The PEGs and PEGs II Suites (Qiagen) and Wizard Classic I–IV (Rigaku Reagents). Protein that had been pre-incubated with ADP was screened against JCSG+ (Jena Bioscience), Optimix4PEG, The PEGs and PEGs II Suites and Wizard Classic I–IV. Crystal formation was prominently observed with ADP in conditions containing Tris and Mg^2+^ salts, prompting a fine screen in a 96-well format using MgCl_2_ and magnesium formate with PEG 400 or PEG 3350 as precipitants, buffered by Tris at pH 8, 8.5 and 9. Formation of the best crystals was observed after six days in a condition consisting of 25% PEG 3350, Tris pH 8.5, 0.4 *M* MgCl_2_ with the protein incubated with ADP at a molar ratio of 1:313. Crystals were collected after ten days and were cryoprotected in reservoir solution supplemented with 20%(*v*/*v*) glycerol. Crystallization information is summarized in Table 2 ▸.

### 2.3. Data collection and processing

Diffraction data were collected on beamline P13 operated by EMBL Hamburg at the PETRA III storage ring, DESY, Hamburg, Germany (Cianci *et al.*, 2017 ▸). Integration was performed using *XDS* (Kabsch, 2010 ▸) and data scaling and merging were carried out with *AIMLESS* (Evans & Murshudov, 2013 ▸) via the *CCP*4*i*2 interface (Potterton *et al.*, 2018 ▸). The relevant statistics are given in Table 3 ▸. Data processing focused on CC_1/2_ as a primary quality parameter, with the goal of achieving a value above 50% (Karplus & Diederichs, 2015 ▸). Consequently, data were processed to a resolution of 1.9 Å, resulting in CC_1/2_ = 0.568 in the highest resolution shell and an 〈*I*/σ(*I*)〉 of 1.28. Data-collection and processing statistics are summarized in Table 3 ▸.

### 2.4. Structure solution and refinement

The structure was determined by molecular replacement using the published crystal structure of the Aurora-A–MYCN complex (PDB entry 5g1x; Richards *et al.*, 2016 ▸) as a search model. Due to the lack of electron density for the terminal regions of the MYCN peptide, it was truncated in the search model to residues 72–89 and molecular replacement was repeated. The mutations at Lys339 of Aurora-A and Asn85 of MYCN and the linker were built in *MOE* (version 2020.09; Chemical Computing Group, Montreal, Canada) and restraints for the maleimidopropionic acid linker were derived using *AceDRG* (Long *et al.*, 2017 ▸). The covalent connections between the maleimidopropionic acid moiety and residues Cys339 of Aurora-A and Lys85 of MYCN were defined during refinement by using geometry restraints in *phenix.refine* (Liebschner *et al.*, 2019 ▸). Parameters were copied from analogous moieties in the *REFMAC* monomer libraries (Supplementary Table S1). The Bicine molecule was fitted to the electron density in *Coot* (Emsley *et al.*, 2010 ▸). Refinement statistics are summarized in Table 4 ▸.

### 2.5. NMR experiments

NMR measurements were performed on a Bruker Avance III 400 MHz spectrometer operating at 400.13 MHz. A PABBI 1H/D-BB Z-GRD inverse probe was used at 300 K and adjusted using a Bruker BSVT temperature-control unit. Pulse sequences were taken from the Bruker pulse-program library with STD-NMR measurements using the *stddiffesgp.*3 program with the saturation frequencies for on and off spectra set to −400 and −16 000 Hz, respectively. Saturation was employed for 4 s with a relaxation time delay of 6 s. The residual water signal was suppressed at 4.703 p.p.m. via the excitation-sculpting method with the time for spin lock to suppress protein signals set to 30 ms. All measurements were conducted in the same buffer conditions (200 m*M* NaCl, 100 m*M* K_2_HPO_4_, 5 m*M* MgCl_2_, 10% glycerol and 3.85% DMSO-d_6_ in D_2_O, pD = 7.0) at 3.85 µ*M* protein concentration and 1 m*M* nucleotide concentration (260-fold excess over the protein). Measurements with kinase inhibitor also included 10 µ*M* MK-5108 (2.6-fold excess over the protein). Data were processed and analysed using the *TopSpin* 4.1.0 software from Bruker.

### 2.6. Molecular-dynamics simulations

The published crystal structure (PDB entry 5g1x) was prepared in *MOE* via the structure-preparation module. Missing side chains were added, ligands were removed and protonation states at pH 9 were determined (*Protonate*3*D*; Labute, 2009 ▸). The cross-link was built and colliding water molecules were removed. Parameters for the cross-link moiety were calculated by extracting the connected lysine and cysteine together with the linker and splitting the molecule at the central amide bond between the lysine and propionic acid. All free ends were capped with ACE (acetyl) or NME (*N*-methyl) groups at the N- and C-terminus, respectively, and partial charges were calculated using *Gaussian*09 Revision C.01 (Frisch *et al.*, 2010 ▸) at the HF/6-31G* level. The *Antechamber* module of *AmberTools*18 (Case *et al.*, 2018 ▸) was used for RESP-fitting and atom-typing compatible with the AMBER ff14sb force field. Next, the *Prepgen* utility was used to create amino-acid templates, and unparameterized di­hedrals in the new templates were compared with the existing ones and modified accordingly. Topology and parameter files for the entire systems were built using the *tleap* module. The complexes were minimized for 2000 steps with an implicit water model (generalized Born implicit solvent model; Tsui & Case, 2000 ▸) using the *pmemd.MPI* module of *AMBER*18. After adding sodium ions for neutralization, the systems were solvated in a box of TIP3P water (Jorgensen *et al.*, 1983 ▸) with a minimum protein–box distance of 10 Å. The simulations were performed using *NAMD* 2.13 (Phillips *et al.*, 2020 ▸). Periodic boundary conditions were applied and electrostatic inter­actions were handled with the particle Ewald methodology (Darden *et al.*, 1993 ▸). System equilibration was carried out by 10 000 steps of minimization, followed by heating from 100 to 300 K over 500 ps at constant volume. Harmonic constraints (0.5 kcal mol^−1^ Å^−2^) were applied to nonsolvent atoms for the first 100 ps and were gradually lowered during the remaining heating process. Afterwards, the systems were allowed to move freely for another 500 ps. Starting from the equilibrated systems, production runs were performed for 100 ns in triplicate for each system at constant pressure (1.01325 bar, Nosé–Hoover Langevin piston pressure control) and constant temperature (300 K, Langevin dynamics). 2 fs timesteps were used for integration and coordinates for output trajectories were saved every 500 steps (1 ps). Structural analysis of the trajectories was performed using *cpptraj* (Roe & Cheatham, 2013 ▸). 2D r.m.s.d. plots were generated for the combined trajectories of a system using every tenth snapshot.

## 3. Results and discussion

### 3.1. Design of the covalently linked Aurora-A–MYCN complex (ANX)

Based on the published crystal structure of the Aurora-A–MYCN complex (PDB entry 5g1x; Richards *et al.*, 2016 ▸), suitable sites for covalently linking the two binding partners were investigated. To avoid any interference with nucleotide or ligand binding, the linkage site should be sufficiently distant from the ATP binding pocket and neighbouring areas. Furthermore, connecting two stable secondary-structure elements was thought to provide a higher chance of establishing a stable link. Accordingly, the contact between α-helix G of Aurora-A and the distal part of the helical substructure of MYCN appeared to be suitable. Specifically, positions 339 of Aurora-A and 85 of MYCN showed the appropriate orientation and distance for linking with a maleimide propionic acid attached to the N^ɛ^ atom of a lysine side chain. Instead of using the lysine that is already present at position 339 in Aurora-A, introduction of a modified lysine into the MYCN peptide was preferred to enable solid-phase synthesis with this unnatural amino acid. As a consequence, the mutation K339C had to be introduced into Aurora-A to enable its ligation to the maleimide head group.

**Figure 2:**
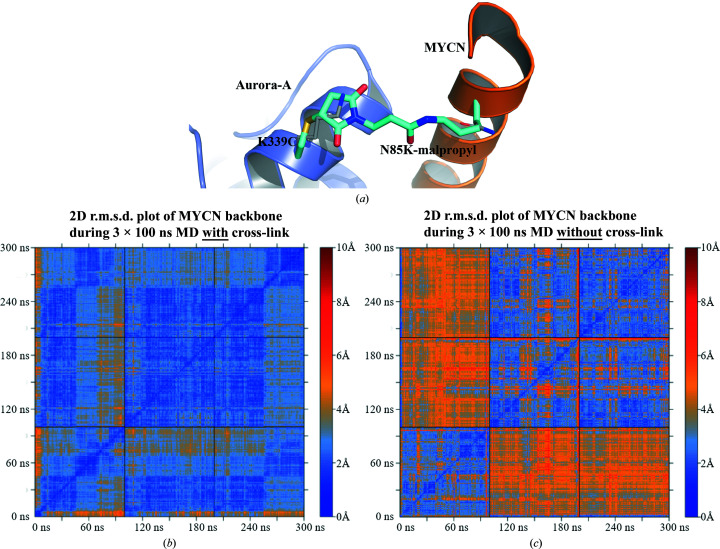
(*a*) Close-up view of the published Aurora-A crystal structure in complex with MYCN (PDB entry 5g1x) and modelled mutations forming a covalent bond (cyan). The original side chains are visible in grey, showing the same orientation as the modelled cross-link moiety. (*b*) 2D r.m.s.d. plot of MYCN backbone atoms during 3 × 100 ns MD simulations of ANX. Each 100 ns square represents an independent simulation compared with the replicates. Similar backbone conformations can be observed during all simulations of the modelled ANX construct. (*c*) 2D r.m.s.d. plot of MYCN backbone atoms during 3 × 100 ns MD simulations without a covalent link being present. The first simulation generates different conformations compared with the second and third simulations, with larger overall deviations in comparison to the simulations of ANX.

To verify the suitability of this construct, the mutations and the covalent link were modelled *in silico* (Fig. 2 ▸ *a*) and molecular-dynamics (MD) simulations were performed on both the *in silico* model of ANX and the unlinked structure. Three independent simulations consisting of 100 ns production runs were carried out for each structure and evaluated based on the r.m.s.d. values of the MYCN backbone atoms after aligning the trajectories on the Aurora-A backbone atoms (Fig. 2 ▸ *b* and 2 ▸ *c*). The observed conformations for the cross-linked construct were stable and very similar in all three trajectories, indicating that the chosen mutations and the length of the cross-link moiety are compatible with a stable Aurora-A–MYCN complex. The results from the unlinked protein–protein complex showed larger deviations between the individual trajectories and more rapid fluctuations within the single trajectories. Hence, we concluded that the proposed mutations would allow the creation of a stabilized Aurora-A–MYCN construct.

### 3.2. Overall structure of ANX and comparison with the structure of the non-cross-linked complex

**Figure 3:**
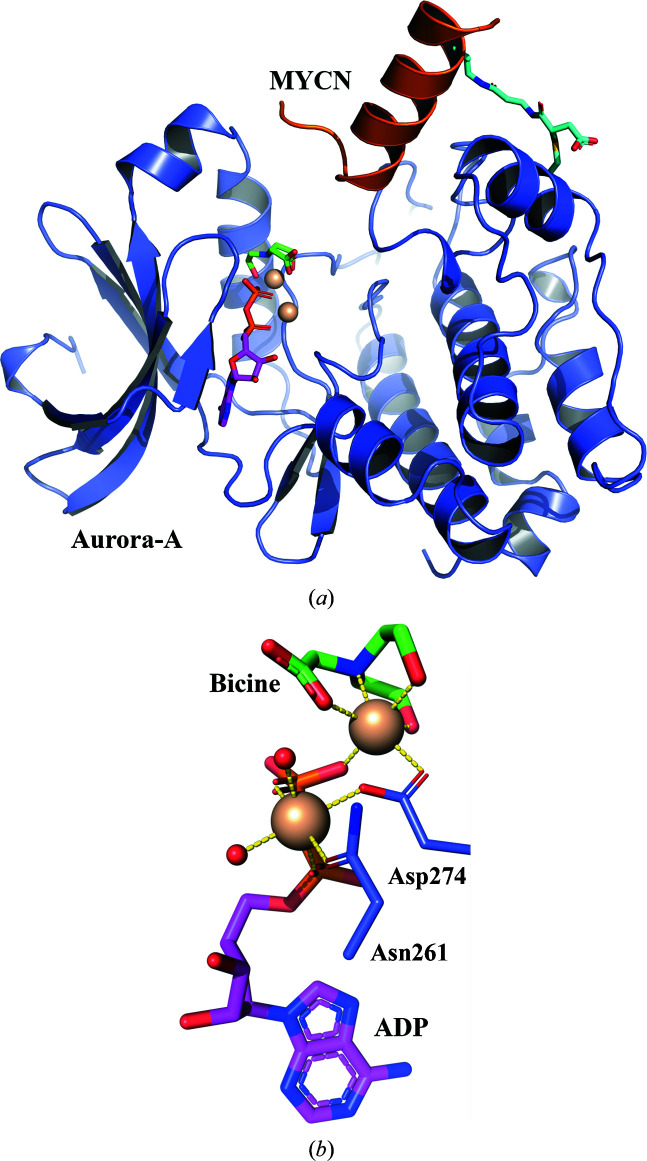
(*a*) Overview of the covalently linked ANX structure (Aurora-A, blue; MYCN, orange; ADP, magenta; Bicine, green; cross-link, cyan). (*b*) Bicine, ADP, Asn261 and Asp274 together with two water molecules coordinate the Mg^2+^ ions in an octahedral fashion.

Cross-linking of Aurora-A and the MYCN peptide was accomplished by incubation at room temperature for 4 h and quenching the reaction by the addition of DTT, as further described in Section 2. The cross-linked construct of Aurora-A and MYCN was crystallized in the presence of ADP. From the resulting crystals, the structure could be determined to a resolution of 1.90 Å (Fig. 3 ▸ *a*). Residues 126–389 of Aurora-A were observed in the electron density, leaving only residues 122–125 and 390–403 at the termini as missing. For MYCN, only residues 72–89 were sufficiently defined in the electron-density maps; this sequence includes the C-terminal helix featuring residue 85, the residue at which the maleimide moiety was introduced.

The protein main chains superimpose well with the published structure of the non-cross-linked Aurora-A–MYCN complex (PDB entry 5g1x), with an r.m.s.d. of 0.21 Å for the main-chain atoms. ADP is coordinated by two Mg^2+^ ions in the nucleotide binding pocket (Fig. 3 ▸ *b*). The first Mg^2+^ ion, referred to as proximal, is octahedrally coordinated by one O atom of each phosphate, the side chains of Asn261 and Asp274, and two water molecules. A Bicine molecule from the storage buffer is coordinated to the second (distal) Mg^2+^ ion, again exhibiting octahedral geometry, with the remaining coordination sites occupied by the side chain of Asp274 and an O atom of the distal phosphate group. The Bicine molecule is present with full occupancy and is well ordered, as reflected by its *B* factors (for individual atoms these vary between 30.4 and 35.7 Å^2^, with an average value of 31.8 Å^2^), which are very similar to the average *B* factor of all surrounding atoms within 5 Å of the ligand (33.7 Å^2^). We also note that the average *B* factor of ADP is almost identical at 31.4 Å^2^ (those for individual atoms vary between 27.1 and 35.9 Å^2^).

### 3.3. Details of the cross-link

**Figure 4:**
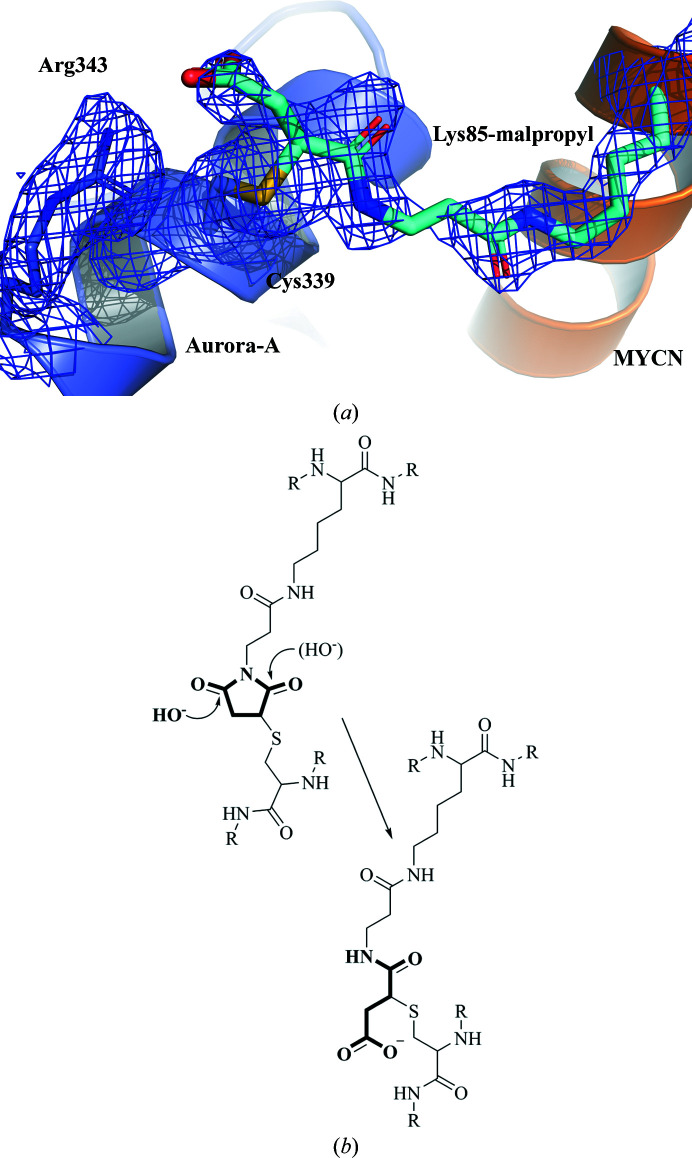
Cross-link between Aurora-A and MYCN. (*a*) 2*F*_o_ − *F*_c_ electron density contoured at 1σ together with a model showing the ring-opened cross-link in cyan, Aurora-A in blue and MYCN in orange. (*b*) Mechanism of base-catalyzed ring hydrolysis. The hydroxyl group can perform a nucleophilic attack at either of the two carbonyl groups. The one depicted in bold is in better agreement with the observed electron density. The second possible attack of a hydroxyl group is shown in parentheses.

The electron-density map clearly shows the modification of Cys339 of Aurora-A. Although the density between Cys339 and Lys85 of MYCN is not perfectly continuous, the propyl maleimide moiety can easily be modelled. The basic conditions (pH 9) of the buffer in which the protein was present prior to crystallization caused the maleimide ring to open, forming a side chain with a free acid function at the end (Fig. 4 ▸). The ring opening of maleimide cross-linking agents is well known and is employed, for example, in antibody–drug conjugates to increase stability (Tumey *et al.*, 2014 ▸). While the closed-ring maleimide cross-link is susceptible to a reverse Michael reaction, the ring-opened form can no longer react back to two individual residues, thus ensuring a stable cross-link. In the ANX structure, the free acid function of the opened maleimide is stabilized by the positively charged side chain of Arg343. Four isomers of the cross-link are possibly created during this process, as the initial attack of the nucleophilic S atom creates a stereocenter in the maleimide ring. In addition, both carbonyl groups can be attacked by a hydroxyl anion, thus generating four different isomers of the ring-opened cross-link in which the N atom of the maleimide and the S^γ^ atom of Cys339 are separated by either two or three C atoms, with the chiral C atom being either in the *S* or the *R* configuration. The resulting electron-density maps suggest that the attack occurs on the carbonyl C atom further away from the S atom. The nonperfect fit of the model into the electron density around Cys339 may, however, reflect the fact that a mixture of the isomers exists in the crystal; despite the relatively high resolution of 1.9 Å, we were unable to convincingly model a mixture of more than one conformation.

### 3.4. Small-molecule binding to ANX

**Figure 5:**
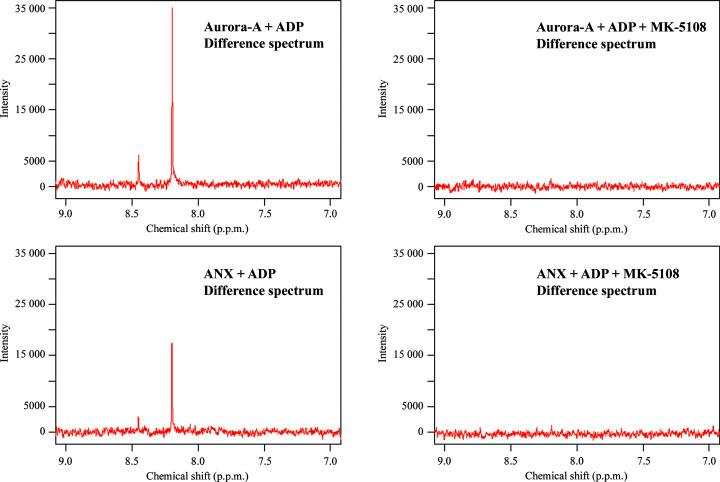
STD-NMR results for Aurora-A and ANX with ADP alone and mixed with MK-5108. On the left, ^1^H peaks at 8.45 and 8.20 p.p.m. can be seen in the difference spectra, which show saturation transfer from the respective protein to ADP. On the right, no saturation difference occurs since MK-5108 blocks the nucleotide binding site. Off-resonance spectra are shown in Supplementary Figs. S1 and S2.

Small-molecule binding to the ANX construct was examined by NMR experiments using ADP and the high-affinity inhibitor MK-5108 (Shimomura *et al.*, 2010 ▸). ADP binding was observed in STD-NMR experiments for both the unlinked Aurora-A mutant and the cross-linked construct. This indicated that the binding pocket of ANX is intact and is not blocked by the unresolved part of the cross-linked peptide. The addition of MK-5108, an Aurora-A inhibitor that displays picomolar affinity (and hence that is not expected to lead itself to any signals in STD-NMR), prevented binding of the nucleotide to both Aurora-A and ANX. This leads to the conclusion that the generated construct is also capable of binding verified Aurora-A inhibitors (Fig. 5 ▸ and Supplementary Figs. S1 and S2).

Rapid hydrolysis of ATP was observed in ^1^H-NMR spectra by comparing the distinguishable signal of H8, the hydrogen atom at position 8 of adenine, in ATP and ADP (Guo *et al.*, 2014 ▸) for both Aurora-A and ANX, thus demonstrating that the cross-linked complex is properly folded and retains not only its ability to bind to small molecules but also its catalytic activity (Supplementary Fig. S3). This will enable screening for small molecules which bind to the Aurora-A–MYCN complex and an iterative structure-guided optimization process. By performing complementary experiments with Aurora-A, hit compounds should be optimizable for selectivity for the complex over the kinase alone. Implementing such selective small molecules, for example, as recognition units for proteolysis-targeting chimeras (PROTACs), would allow the selective degradation of the Aurora-A–MYCN complex and therefore target the undruggable transcription factor.

In summary, we have designed, synthesized and characterized a covalently linked Aurora-A–MYCN complex, which is suitable for binding experiments, screening for small-molecule binders with *in vitro* assays and, ultimately, crystal structure determination.

## Supplementary Material

PDB reference: covalently linked Aurora-A–MYCN complex, 7ztl

Supplementary Table and Figures. DOI: 10.1107/S2059798322011433/ud5038sup1.pdf

## Figures and Tables

**Table 1:** Macromolecule-production information

Source organism	*Homo sapiens*
DNA source	pETM11-AURKA 122–403 wt
Forward primers	5′-catcaaaaccatcaaatGCCcaaaacaaagaatcagctagcaaacag-3′
	5′-ccaggaggaccactctcGCTggcaccctggactacctg-3′
	5′-cataccaagagacctacTGTagaatatcacgggttgaattcacattc-3’
Reverse primers	5′-aggtagtccagggtgccAGCgagagtggtcctcctggaag-3′
	5′-tcaacccgtgatattctACAgtaggtctcttggtatgtgtttgc-3′
	5′-gctgattctttgttttgGGCatttgatggttttgatgaatttgctgtg-3′
Cloning vector	pETM11
Expression vector	pETM11
Expression host	*E. coli* BL21(DE3) pLysS (Novagen)
Complete amino-acid sequence of the construct produced	MKHHHHHHPMSDYDIPTTENLYFQGAMESKKRQWALEDFEIGRPLGKGKFGNVYLAREKQSKFILALKVLFKAQLEKAGVEHQLRREVEIQSHLRHPNILRLYGYFHDATRVYLILEYAPLGTVYRELQKLSKFDEQRTATYITELANALSYCHSKRVIHRDIKPENLLLGSAGELKIADFGWSVHAPSSRRTTLAGTLDYLPPEMIEGRMHDEKVDLWSLGVLCYEFLVGKPPFEANTYQETYCRISRVEFTFPDFVTEGARDLISRLLKHNPSQRPMLREVLEHPWITANSSKPSNAQNKESASKQS

**Table 2:** Crystallization

Method	Sitting-drop vapour diffusion
Plate type	96-well plates
Temperature (K)	293
Protein concentration (mg ml^−1^)	3.44
Buffer composition of protein solution	0.1 *M* Bicine pH 9, 0.25 *M* NaCl, 5 m*M* MgCl_2_, 10% glycerol
Composition of reservoir solution	0.1 *M* Tris pH 8.5, 0.4 *M* MgCl_2_, 25% PEG 3350
Volume and ratio of drop (nl)	400 (1:1)
Volume of reservoir (µl)	50

**Table 3:** Data collection and processing Values in parentheses are for the outer shell.

Diffraction source	Beamline P13, EMBL Hamburg
Wavelength (Å)	0.976246
Temperature (K)	100
Detector	EIGER 6M
Crystal-to-detector distance (mm)	253.06
Rotation range per image (°)	0.1
Total rotation range (°)	360
Exposure time per image (s)	0.008
Space group	*P*3_2_21
*a*, *b*, *c* (Å)	86.61, 86.61, 91.79
α, β, γ (°)	90, 90, 120
Mosaicity (°)	0.06
Resolution range (Å)	39.17–1.90 (1.97–1.90)
Total No. of reflections	630894 (63615)
No. of unique reflections	31825 (3148)
Completeness (%)	99.94 (99.84)
Multiplicity	19.8 (20.2)
〈*I*/σ(*I*)〉	11.06 (1.28)
*R*_merge_ †	0.1551 (2.516)
*R*_p.i.m_ ‡	0.03537 (0.5728)
CC_1/2_ §	0.998 (0.568)
Overall *B* factor from Wilson plot (Å^2^)	39.88

† *R*_merge_ = 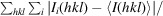 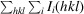 .

‡ *R*_p.i.m_ = 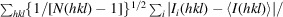 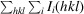 .

§ CC_1/2_ = 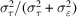 .

**Table 4:** Structure solution and refinement Values in parentheses are for the outer shell.

Resolution range (Å)	39.17–1.90 (1.97–1.90)
Completeness (%)	99.94 (99.84)
No. of reflections, working set	31811 (3146)
No. of reflections, test set	1594 (164)
Final *R*_cryst_ †	0.1637 (0.3007)
Final *R*_free_ †	0.1934 (0.3350)
Coordinate error (Å)	0.25
No. of non-H atoms	
Protein	2319
Ions	3
Ligands	68
Waters	210
Total	2600
R.m.s. deviations	
Bond lengths (Å)	0.008
Bond angles (°)	1.03
Average *B* factor (Å^2^)	
Overall	44.57
Protein	45.07
Ligand	45.66
Water	52.76
Ramachandran plot	
Most favoured (%)	96.73
Allowed (%)	2.91
Outliers (%)	0.36
PDB code	7ztl

† *R* = 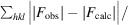 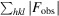 , where *R*_free_ was calculated with 5% of the data that were omitted from refinement.
