# Supplementary material for: Crystal structure of a covalently linked Aurora-A–MYCN complex
Source: Acta Crystallogr D Struct Biol. 2023 Jan 1;79(Pt 1):1–9. doi: 10.1107/S2059798322011433 (PMC9815099; doi:10.1107/S2059798322011433)
Supplement: Supplementary file 1 [file d-79-00001-sup1.pdf]

STRUCTURAL BIOLOGY

Volume 78 (2022)

Supporting information for article:

Crystal structure of a covalently linked Aurora-A–MYCN complex

Mathias Diebold, Lars Schönemann, Martin Eilers, Christoph Sotriffer and Hermann Schindelin

Table S1 Geometry restraints used during refinement with phenix.refine. Ideal and sigma values were taken from the Refmac monomer library.

Bonds	Distance	Sigma
(chain ‘B’ and (resid 90 and (name C1)))<br>(chain ‘B’ and (resid 85 and (name NZ)))	1.321	0.02
(chain ‘A’ and (resid 339 and (same SG)))<br>(chain ‘B’ and (resid 90 and (name C5)))	1.811	0.02
**Angles**	**Angle**	**Sigma**
(chain ‘A’ and (resid 339 and (name CB)))<br>(chain ‘A’ and (resid 339 and (name SG)))<br>(chain ‘B’ and (resid 90 and (name C5)))	100.595	1.5
(chain ‘B’ and (resid 90 and (name O1)))<br>(chain ‘B’ and (resid 90 and (name C1)))<br>(chain ‘B’ and (resid 85 and (name NZ)))	123.5	3
(chain ‘B’ and (resid 90 and (name C4)))<br>(chain ‘B’ and (resid 90 and (name C5)))<br>(chain ‘A’ and (resid 339 and (name SG)))	109.47	1.5
(chain ‘B’ and (resid 90 and (name C6)))<br>(chain ‘B’ and (resid 90 and (name C5)))<br>(chain ‘A’ and (resid 339 and (name SG)))	109.47	1.5
(chain ‘B’ and (resid 85 and (name CE)))<br>(chain ‘B’ and (resid 85 and (name NZ)))<br>(chain ‘A’ and (resid 90 and (name C1)))	122	3
**Planes**		**Sigma**
(chain ‘B’ and ((resid 85 and (name CE or name NZ)) or (resid 90 and (name C1 or name C2 or name O1)))		0.02

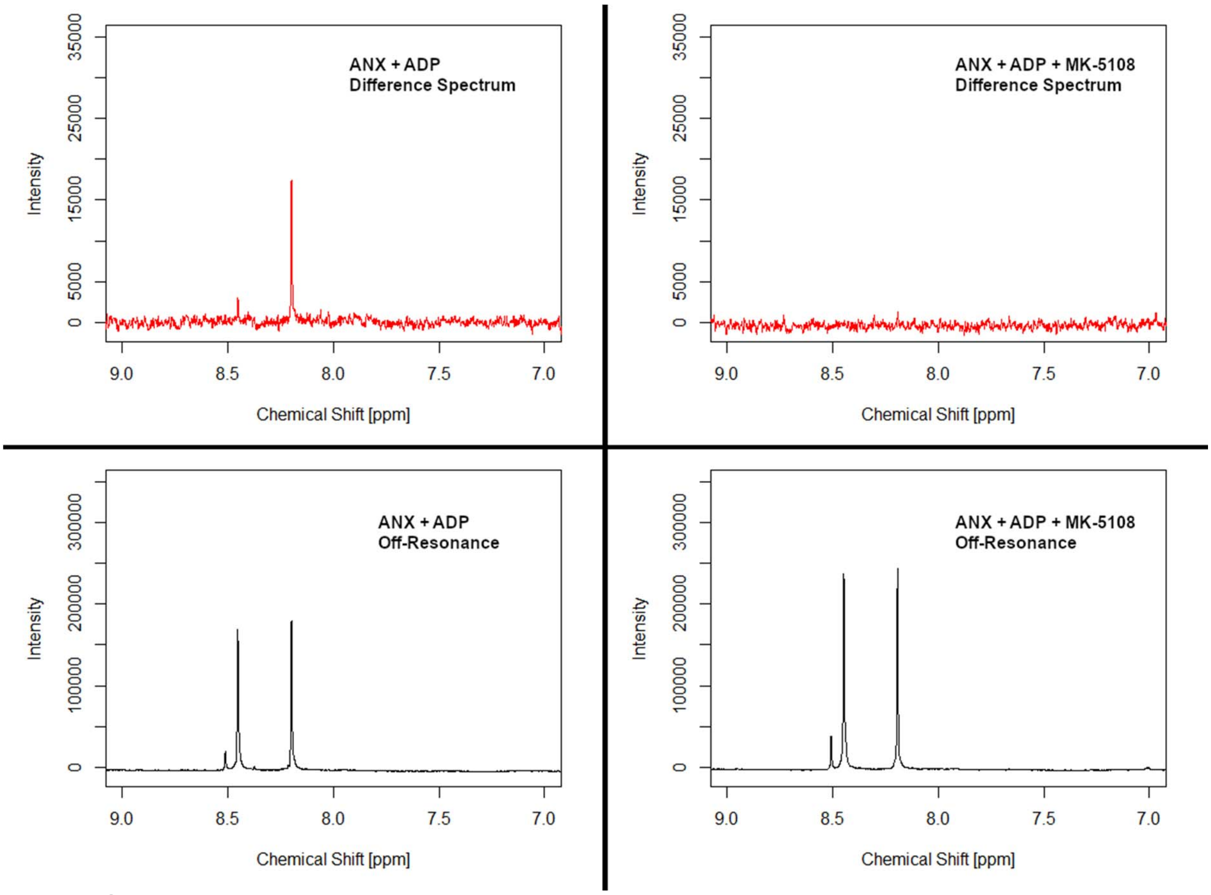

Figure S1 STD-NMR results of ANX with ADP alone and mixed with MK-5108. Off-resonance spectra show that the ^{1}H-peaks of ADP are present in each assay. The difference spectra demonstrate that a saturation transfer only occurs in the absence of MK-5108.

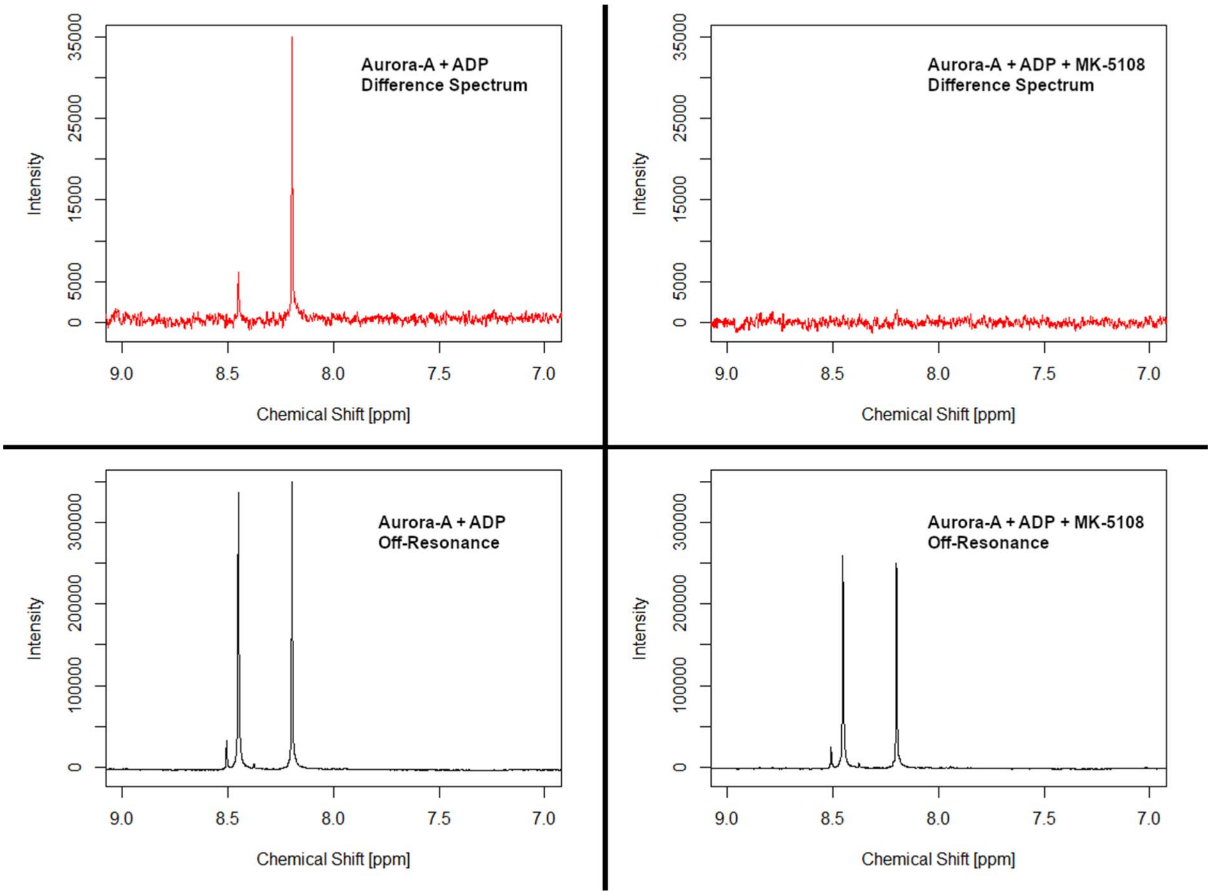

Figure S2 STD-NMR results of Aurora-A with ADP alone and mixed with MK-5108. Off-resonance spectra show that the ^{1}H-peaks of ADP are present in each assay. The difference spectra document that a saturation transfer only occurs in the absence of MK-5108.

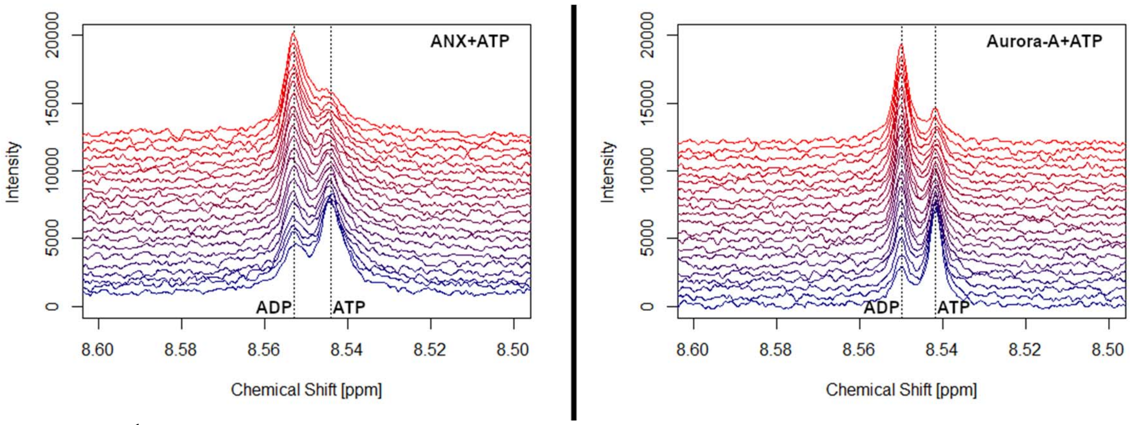

Figure S3 ^{1}H-NMR results of Aurora-A and ANX with ATP. The time series extends from the lowest (blue) spectrum to the top (red) with an intensity off-set of 600. The time between measurements varied between 133 and 134 s. The first measurement of ANX with ATP started 510 s after mixing, the first measurement of Aurora-A with ATP after 480 s.
